# Distribution and seasonal differences in Pacific Lamprey and *Lampetra* spp eDNA across 18 Puget Sound watersheds

**DOI:** 10.7717/peerj.4496

**Published:** 2018-03-16

**Authors:** Carl O. Ostberg, Dorothy M. Chase, Michael C. Hayes, Jeffrey J. Duda

**Affiliations:** Western Fisheries Research Center, U.S. Geological Survey, Seattle, WA, United States of America

**Keywords:** Environmental DNA, Locked nucleic acids, Quantitative PCR, Conservation, Puget sound, Pacific Lamprey, *Lampetra*, *Entosphenus*

## Abstract

Lampreys have a worldwide distribution, are functionally important to ecological communities and serve significant roles in many cultures. In Pacific coast drainages of North America, lamprey populations have suffered large declines. However, lamprey population status and trends within many areas of this region are unknown and such information is needed for advancing conservation goals. We developed two quantitative PCR-based, aquatic environmental DNA (eDNA) assays for detection of Pacific Lamprey (*Entosphenus tridentatus*) and *Lampetra* spp, using locked nucleic acids (LNAs) in the probe design. We used these assays to characterize the spatial distribution of lamprey in 18 watersheds of Puget Sound, Washington, by collecting water samples in spring and fall. Pacific Lamprey and *Lampetra* spp were each detected in 14 watersheds and co-occurred in 10 watersheds. Lamprey eDNA detection rates were much higher in spring compared to fall. Specifically, the Pacific Lamprey eDNA detection rate was 3.5 times higher in spring and the *Lampetra* spp eDNA detection rate was 1.5 times higher in spring even though larval lamprey are present in streams year-round. This significant finding highlights the importance of seasonality on eDNA detection. Higher stream discharge in the fall likely contributed to reduced eDNA detection rates, although seasonal life history events may have also contributed. These eDNA assays differentiate Pacific Lamprey and *Lampetra* spp across much of their range along the west coast of North America. Sequence analysis indicates the Pacific Lamprey assay also targets other *Entosphenus* spp and indicates the *Lampetra* spp assay may have limited or no capability of detecting *Lampetra* in some locations south of the Columbia River Basin. Nevertheless, these assays will serve as a valuable tool for resource managers and have direct application to lamprey conservation efforts, such as mapping species distributions, occupancy modeling, and monitoring translocations and reintroductions.

## Introduction

Pacific Lamprey (*Entosphenus tridentatus*) were historically distributed throughout North Pacific drainages from Mexico to Japan, and Western Brook Lamprey (*Lampetra richardsoni*) and Western River Lamprey (*L. ayresii*) were historically distributed throughout Pacific coast drainages of North America (Potter et al., 2015). Growing concern for the status of Pacific Lamprey, Western Brook Lamprey and Western River Lamprey populations across Pacific coast drainages of North America led to a petition calling for their listing under the Endangered Species Act in 2003 (Nawa et al., 2003). The US Fish and Wildlife Service (USFWS) ruled that these species were ineligible for listing in part because information on population status and trends were lacking (USFWS, 2004).

The cultural importance of Pacific Lamprey to Native American tribes and the increased awareness of conservation needs and challenges resulted in the Pacific Lamprey Conservation Initiative (PLCI). The goal of the initiative is to improve the range wide status of Pacific Lamprey through coordinated efforts among federal, tribal, and state agencies (Luzier et al., 2011; Wang & Schaller, 2015). The initial PLCI population assessment found that Pacific Lamprey have declined throughout their range and a risk analysis determined many populations are imperiled or may be extirpated (Luzier et al., 2011). Furthermore, risk analysis for Pacific Lamprey in much of Western Washington was not performed as part of this assessment because regional information on their abundance and distribution was lacking. The lack of information on population status hinders Pacific Lamprey conservation and restoration efforts. A key research and monitoring need is determining the distribution, occupancy status and population trends of Pacific Lamprey (Clemens et al., 2017).

Lamprey have a complex life history. Ammocoetes (hereafter, larvae) reside in sediments in freshwater as filter feeders for 3–7 years after which they metamorphose into juveniles (Dawson et al., 2015). Depending on the species, juveniles exhibit a parasitic or non-parasitic life history strategy. Parasitic lampreys feed on host fish in fresh and saltwater for 1–2 years prior to sexual maturation (Manzon, Youson & Holmes, 2015). Non-parasitic lampreys begin sexual maturation almost immediately after metamorphosis and consequently stop feeding following metamorphosis (Docker, 2009). Specifically, Pacific Lamprey and Western River Lamprey are anadromous and parasitic, whereas Western Brook Lamprey reside in freshwater habitats and are non-parasitic (Potter et al., 2015). Adults of all species spawn in freshwater and are semelparous (die after spawning).

Aquatic environmental DNA (eDNA) is a rapidly emerging tool that has application to conservation biology and ecology (Barnes & Turner, 2016; Rees et al., 2014b; Thomsen & Willerslev, 2015). eDNA methods are based on the premise that tissues and cells containing DNA are shed into the aquatic environment from source organisms through feces, skin, gills, gametes, and decomposing individuals. Therefore, eDNA can be extracted from water samples, allowing target species residing in aquatic habitats to be detected through genetic markers via PCR methods or direct sequencing. Studies on eDNA transport in flowing water indicate that eDNA from some target species may be detected only over short distances from their source (less than 50 m) (Pilliod et al., 2014), whereas eDNA from other target species may be detected over hundreds of meters to several kilometers from their source (Balasingham, Walter & Heath, 2017; Deiner & Altermatt, 2014; Jane et al., 2015). eDNA has been used to detect invasive (Dejean et al., 2012; Goldberg et al., 2013) and imperiled species (De Souza et al., 2016; Laramie, Pilliod & Goldberg, 2015; McKelvey et al., 2016), as well as to explore community structure (Kelly et al., 2014; Port et al., 2016). eDNA-based methods can be effective for identifying species that are otherwise difficult to detect with traditional sampling methods and may have increased detection probabilities relative to traditional sampling methods (Dejean et al., 2012; Jerde et al., 2011; Pilliod et al., 2013), even when species occur at low densities (Pilliod et al., 2013). Thus, eDNA is an ideal tool for monitoring the distribution of aquatic species that have an extended freshwater rearing phase or are patchily distributed, such as lamprey (Torgersen & Close, 2004).

**Figure 1:**
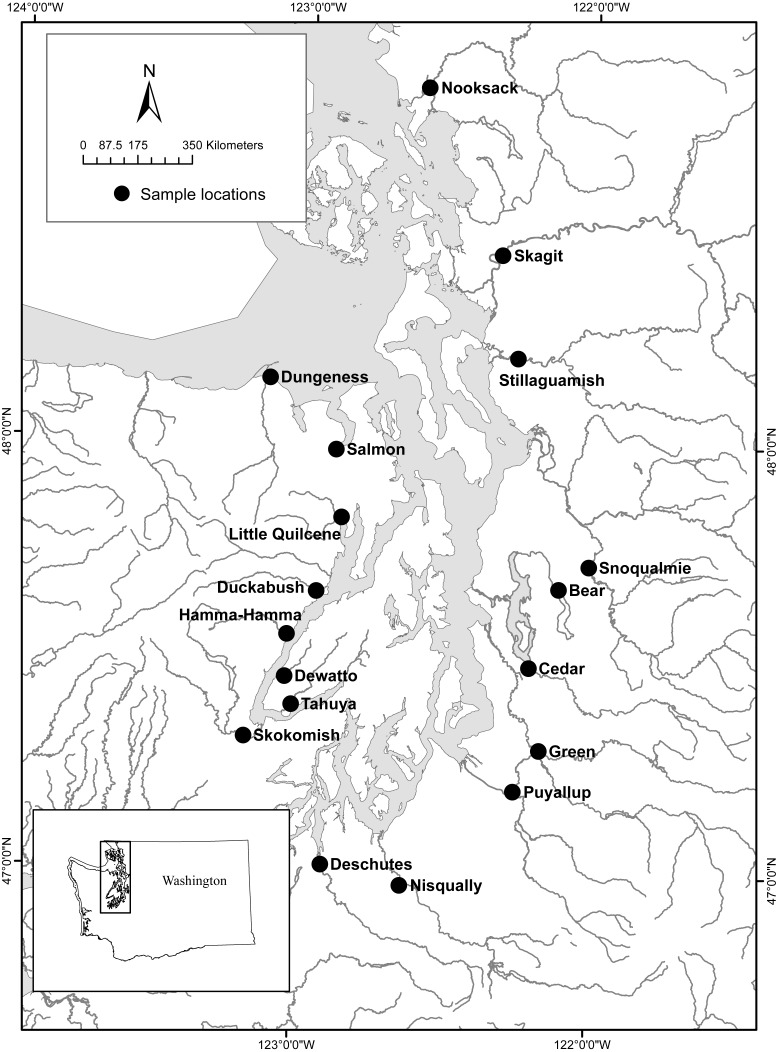
Study area and eDNA collection sites for 18 Puget Sound watersheds (figure modified from Hayes et al. (2013)).

We had two objectives for this research study. The first was to develop two lamprey eDNA assays, one that targets Pacific Lamprey and one that targets both Western River Lamprey and Western Brook Lamprey (i.e., *Lampetra* spp). We developed an eDNA assay inclusive for Western River and Western Brook Lamprey because these two species are closely related, form a single clade, and are difficult to differentiate genetically (Boguski et al., 2012). Therefore, we refer to Western Brook and Western River Lamprey as *Lampetra* spp, hereafter, unless noted otherwise. The second objective of this study was to use the Pacific Lamprey and the *Lampetra* spp eDNA assays to survey 18 watersheds of Puget Sound, Washington, (Fig. 1) for the presence of Pacific Lamprey and *Lampetra* spp, and compare seasonal differences in eDNA concentration (spring vs. fall). The presence of Pacific Lamprey and *Lampetra* spp in these 18 watersheds was previously identified by Hayes et al. (2013) by using incidental captures of lamprey in traps designed for sampling outmigrating juvenile salmonids, which provided a benchmark for comparison with existing data.

## Materials & Methods

### Pacific Lamprey and *Lampetra* spp quantitative PCR assay development

#### Genomic DNA extraction and sequence analysis

We used DNeasy kits (Qiagen, Valencia, CA, USA) to extract fin tissue DNA from 13 Pacific Lamprey and 23 *Lampetra* spp collections representing several Puget Sound watersheds (Table S1). These lamprey were identified as Pacific Lamprey and *Lampetra* spp in Hayes et al. (2013) by using morphological and genetic methods. We sequenced 886 bp of the mitochondrial DNA cytochrome b (cytb) gene from each individual by using the primers Ltr-Cytb-F, 5′-CCTGATGAAATTTTGGCTCAC-3′, and Ltr-Cytb-R, 5′-CACTGTTGATGCAACTTGTGTT-3′, developed using sequence data from Boguski et al. (2012). PCR amplifications were performed in 20 µL reaction volumes, consisting of 15 ng genomic DNA, 1X NH4 Reaction Buffer (Bioline, London, UK), 2.5 mM MgCl_2_, 200 μM each dNTP (Bioline, London, UK), 150 nM of each primer, and 0.5 units *Taq* DNA polymerase (Bioline, London, UK). Cycling conditions consisted of 95 °C for 2 min, followed by 35 cycles of 95 °C for 15 s, 62 °C for 1 min, and 72 °C for 1 min. PCR products were sequenced using a 3730xl DNA Analyzer (Applied Biosystems, Foster City, CA, USA) and sequences were edited and aligned using SEQUENCHER v 4.10.1 (Gene Codes Corporation, Ann Arbor, MI, USA). All sequences were deposited into GenBank (KU672473–KU672508).

#### Quantitative PCR assay design and protocol

The Puget Sound lamprey cytb sequence data was used to develop a Pacific Lamprey-specific (ETCytb_890-1015) and a *Lampetra* spp-specific (LACytb_890-1015) quantitative PCR (qPCR) assay. Each assay incorporated locked nucleic acids (LNAs) into the probe design (Table 1). LNAs are nucleotide analogs which contain a methylene bridge between the 2′-oxygen and 4′-carbon of the ribose group, locking the ribose group into a rigid bicyclic formation (Braasch & Corey, 2001). This conformational change enhances hybridization of LNA containing probes to complimentary single stranded DNA, improves mismatch discrimination, and increases thermal stability of duplex complementary DNA (Koshkin et al., 1998; Owczarzy et al., 2011; Petersen et al., 2000; Vester & Wengel, 2004; You et al., 2006). The assays (TaqMan LNA assays) were designed by Integrated DNA Technologies and the probes were designed using methods described by Johnson, Haupt & Griffiths (2004). The ETCytb_890-1015 and LACytb_890-1015 assays target the same 126 bp cytb region in Pacific Lamprey and *Lampetra* spp. The probe sequences have a 5 bp mismatch between Puget Sound *Lampetra* spp and Pacific Lamprey and we incorporated LNAs at four of the mismatches. Probes were labeled with 6-FAM at the 5′ end, ZEN as an internal quencher, and Iowa Black FQ on the 3′ end. The forward primer sequence is conserved and the reverse primer sequence differs by one bp between Puget Sound Pacific Lamprey and *Lampetra* spp.

**Table 1:** Primer and probe sequences for the Pacific Lamprey (ETCytb_890-1015) and *Lampetra* spp (LACytb_890-1015) qPCR assays developed in this study.

Target taxa	Forward primer	Reverse primer	Probe^a^
Pacific Lamprey	CTTTAGCAGCAGCCATCATA	GTAGTGCTAGATCAGCGATTAGAA	TAT + CCAATT + CCG + CCC + AC
*Lampetra* spp	CTTTAGCAGCAGCCATCATA	GTAGTGCTAGATCAGCAATTAGAA	CAT + TCAATT + TCG + TCC + GC

**Notes.**

a Locked nucleic acid nucleotides = +A, +C, +G, +T.

We performed qPCRs using the ViiA 7 real-time PCR System (Applied Biosystems, Foster City, CA, USA) and analyzed results using ViiA 7 RUO 1.2.4 software. Pacific Lamprey and *Lampetra* spp qPCR assays were performed in quadruplicate in 14 μL volumes containing 1x Gene Expression Master Mix (Invitrogen, Carlsbad, CA, USA), 0.7 μM forward primer and 0.7 µM respective reverse primer, 0.2 μM of the respective LNA probe, and 6 μL of DNA template. Cycling conditions for all reactions consisted of 15 min initial heat activation at 95 °C, followed by 50 cycles of denaturing at 95 °C for 1 min and annealing/extension at 60 °C for 1 min. In addition, two oligonucleotide ultramers (Integrated DNA Technologies, Coralville, IA, USA), one consisting of the Pacific Lamprey amplicon and one consisting of the *Lampetra* spp amplicon, were used as standards. To estimate the mtDNA copy number in eDNA samples, we included a five-point standard curve (6 × 10^0^–6 × 10^4^ copies/reaction) for both the Pacific Lamprey and *Lampetra* spp amplicon on each qPCR plate. A no template control (sterile water in place of DNA) was included on each qPCR plate.

### Sensitivity, specificity and validation of lamprey qPCR assays

The limit of detection (LOD) and limit of quantification (LOQ) for the ET_Cytb890-1015 and LACytb_890-1015 assays were determined by performing qPCR on a dilution series of each target oligonucleotide ultramer (1, 2, 3, 6, 30, 60, 300, and 600 copies/qPCR), with eight qPCR replicates per concentration. We regarded LOD as the minimum number of copies that could be detected and we regarded LOQ as the lowest number of copies that yielded positive amplification across the eight replicates (Doi et al., 2017; Takahara, Minamoto & Doi, 2013; Treguier et al., 2014). With these criteria, the LOD was determined as 1 copy/qPCR and the LOQ was determined as six copies/qPCR for each assay (Fig. S1).

To determine the specificity of the ETCytb_890-1015 and LACytb_890-1015 qPCR assays, we performed three tests. First, we tested for cross amplification and reduction of qPCR efficiency in samples containing both Pacific Lamprey and *Lampetra* spp genomic DNA. This goal was accomplished by testing serial dilutions (0.006 pg/μL–600 pg/μL) of Pacific Lamprey DNA in the presence of 1 pg/μL of *Lampetra* spp DNA and vice-versa.

Second, each assay was tested on genomic DNA from 90 Pacific Lamprey and 54 *Lampetra* spp (including *L*. *ayresii*, *L*. *richardsoni*, and *L*. *hubbsi*) distributed from Alaska to California (Table S2), including 13 Pacific Lamprey and 17 *Lampetra* spp from Puget Sound that were originally sequenced, and on genomic DNA from 39 non-target fish species commonly found in the Pacific Northwest (one individual/species) (Table S2). We used 1 ng DNA per qPCR for this test. The threshold cycle (*C*_*t*_) value for each lamprey sampled outside the Puget Sound was compared to the range of *C*_*t*_ values for Pacific Lamprey and *Lampetra* spp sampled from within the Puget Sound watershed because higher *C*_*t*_ values from samples that have no sequence information relative to *C*_*t*_ values from samples with sequence information could indicate basepair mismatches within the primers and/or probe.

Third, ETCytb_890-1015 and LACytb_890-1015 primer and probe sequences were compared to published *Entosphenus* spp and *Lampetra* spp cytb sequence data sampled from Pacific coast drainages of North America (Boguski et al., 2012; Lang et al., 2009; Reid et al., 2011). We retrieved 156 cytb DNA sequences from GenBank and aligned them to the Puget Sound Pacific Lamprey and *Lampetra* spp sequences by using the program MEGA ver. 6 (Tamura et al., 2013). A summary of samples included in the sequence analysis are as follows: Pacific Lamprey, *N* = 24 (13 from our study, 10 from Boguski et al. (2012), and one from Lang et al. (2009)); *E*. *lethophagus*, *E*. *macrostomus*, *E*. *minimus*, and *E*. *similus*, N = one each from Lang et al. (2009); *Lampetra* spp (including *L*. *ayresii*, *L*. *richardsoni*, *L*. *pacifica*, and *L*. *hubbsi*), *N* = 164 (23 from our study, 135 from Boguski et al. (2012), three from Lang et al. (2009), and three from Reid et al. (2011)). In addition, primer and probe sequences were subjected to BLAST and Primer-BLAST analysis against non-target Actinopterygii (ray-finned fishes) as an additional test for specificity. The BLAST and Primer-BLAST analyses against non-target Actinopterygii species did not return any highly homologous matches.

To validate the functionality of our qPCR assays for detecting lamprey DNA in water samples collected in the field, we collected water from flow-through tanks that contained Pacific Lamprey (Umatilla Hatchery, Irrigon, OR, USA) and from a site downstream of a juvenile salmonid fish trap on Bear Creek, Washington, when *Lampetra* spp were known to be present in the trap. Water samples were filtered and tested by the qPCR assays following the methods described herein and results showed that only Pacific Lamprey and *Lampetra* DNA were detected in each respective water sample.

### Detection of lamprey eDNA in Puget Sound watersheds

#### Field collections

To characterize the spatial distribution of lamprey eDNA in Puget Sound watersheds, we collected sub-surface water samples from 18 watersheds (Fig. 1, Table S3) that were known to contain Pacific Lamprey and/or *Lampetra* spp (Hayes et al., 2013). All water samples were collected within 250 m of the location of traps that were monitored by Hayes et al. (2013) and water samples were not targeted toward a specific type of lamprey habitat. Twelve of the 18 watersheds also have stream flow gages operated by the U.S. Geological Survey, and we recorded the median 24 h stream flow for these watersheds on each day that water samples were collected (Table S3). Three 1-L surface water sample replicates (field replicates) were collected from a single location in each watershed between October 9 and November 13, 2014, (hereafter, fall sample collection) and between May 6 and June 30, 2015, (hereafter spring sample collection) and each watershed was visited once during each collection period (fall and spring). Water samples were collected in fall and spring to compare eDNA detection between seasons. Water samples were stored on ice until filtration, which occurred 6–18 h post collection. Water samples were filtered through a pre-sterilized 47 mm diameter filter funnel with a 0.45 um pore size cellulose nitrate sterile filter membrane (Thermo Fisher Scientific, Waltham, MA, USA). One liter of distilled water was also filtered at each filtration session and used as a filtration control in the qPCR to ensure detection of any field contamination. Following the water filtration process, filters were removed from the funnel, placed into sterile 2 mL tubes containing 100% ethanol and stored at −20 °C until DNA extraction. We sterilized all filter funnels, bottles, and forceps prior to their use by soaking in 10% bleach for 10 min followed by rinsing in deionized water.

#### DNA extraction from water samples

Each filter was cut into eight 2 mm strips with sterile scissors prior to DNA extraction, filters were incubated in Lysis buffer for one hour at 55 °C, and DNA embedded on filters was extracted using Qiagen DNeasy spin kits (Qiagen, Valencia, CA, USA) with the following modifications: 360 μL ATL buffer and 40 μL of Proteinase K were used for cell lysis and the volume of AL buffer and 100% ethanol was adjusted to 400 μL post lysis. DNA was eluted into 200 μL of AE buffer and stored at −20 °C until qPCR analysis. To minimize contamination, DNA was extracted in a room where no amplified PCR products or highly concentrated target DNA sequences are allowed. In addition, a negative extraction control (extraction buffers only) was included in the extraction procedure to identify any contamination of equipment and reagents during this procedure.

#### Testing PCR inhibition

All eDNA extracts were tested first for PCR inhibition by performing an internal positive control (IPC) assay using TaqMan Exogenous Internal Positive Control Reagents (EXO-IPC) (Applied Biosystems, Foster City, CA, USA). To test for PCR inhibition, three replicate qPCRs were run for each eDNA sample and the no template control (NTC). Each IPC qPCR consisted of 6 μL of Gene Expression Master Mix , 1.2 µL EXO-IPC mix, 0.24 μL of EXO-IPC DNA, and either 4 μL of template DNA or 4 μL of sterile water for the NTC in a 12 μL total reaction volume. Cycling conditions for the IPC were the same as for the lamprey assays. We considered environmental samples to be inhibited when the mean IPC *C*_*t*_ value for a sample was three or more *C*_*t*_ higher than the mean *C*_*t*_ value for the NTC. No instances of PCR inhibition were found in our environmental samples.

#### qPCR of field collections

The Pacific Lamprey and *Lampetra* spp qPCR assays were performed in quadruplicate on each water sample, thus 12 qPCRs were performed per sample location in the fall and in the spring (3 water samples/location × 4 qPCRs/water sample = 12 qPCRs/location). The ETCytb_890-1015 and LACytb_890-1015 qPCR assays were performed following the methods described herein.

To verify that field collected water samples testing positive for Pacific Lamprey and *Lampetra* spp contained the assay target sequences, we sequenced a portion of the cytb gene region comprising the primers and probes using DNA extracted from water samples collected from Cedar River and Dungeness River in spring, 2015. This goal was accomplished by using the original sequencing primers (Ltr-Cytb-F and Ltr-Cytb-R) to amplify DNA extracted from the water samples, using the PCR protocol described herein for these sequencing primers. Next, a second PCR was performed on the original PCR product, using Ltr-Cytb-R and a nested PCR primer Ltr-Cytb-F1, 5′-GGGCAAATATCCTTCTGAGG-3′, following the PCR protocol described for the sequencing primers. The amplicon from the second PCR was gel purified using a QIAquick Gel Purification Kit (Qiagen, Valencia, CA, USA) and sequenced on a 3130 Genetic Analyzer (Applied Biosystems, Foster City, CA, USA). Sequences were read using SEQUENCHER v 4.10.1 (Gene Codes Corporation, Ann Arbor, MI, USA) and it was confirmed that the Cedar River water sample contained the *Lampetra* spp target sequences and the Dungeness River water sample contained the Pacific Lamprey target sequences, as expected (see ‘Results’).

#### Data analysis

Our criteria for inferring species presence at a specific location at one sample collection (fall or spring) was a minimum of two qPCR amplifications exceeding the LOD (1 copy/qPCR), or one qPCR amplification exceeding the LOD in the original sample analysis and at least one qPCR amplification exceeding the LOD in a repeated sample analysis (sensu Goldberg et al., 2016). For example, when the three field replicate water samples yielded only one qPCR amplification above the LOD, out of the 12 qPCRs performed for a target species, we performed repeat qPCRs across the three field replicates to determine whether the original result was reproducible (i.e., yielded a qPCR amplification that exceeded the LOD). We considered a reproducible result as species presence.

The mean eDNA concentration for Pacific Lamprey and *Lampetra* spp for each sample location was estimated by averaging the eDNA concentration across the 12 qPCRs within each sample collection period. The eDNA concentration for each qPCR that exceeded the LOQ (6 copies/qPCR) was estimated from the standard curve. For qPCRs that exceeded the LOD but did not exceed the LOQ, we assigned a quantity of half the LOQ (3 copies/qPCR) following the method of Klymus et al. (2015), because DNA concentrations below the LOQ cannot be accurately estimated, even though target DNA is detected (i.e., above LOD). We considered the eDNA concentration for qPCRs that failed to amplify or did not exceed the LOD as being zero and included these qPCRs when estimating the mean eDNA concentration.

The eDNA detection rate for fall and for spring was estimated as the frequency of 1-L water samples that tested positive for lamprey eDNA (i.e., exceeded the LOD) averaged across all locations where lamprey eDNA was detected. The eDNA survey results were compared to the results from a study that identified the distribution of lamprey in Puget Sound watersheds (Hayes et al., 2013).

## Results

### Sensitivity, specificity, and validation of Pacific Lamprey and *Lampetra* spp qPCR assays

The PCR efficiency of the Pacific Lamprey *and Lampetra* spp qPCR assay was each greater than 90% and *r*^2^ was greater than 0.99 (Table 2). When qPCR was performed on samples that contained genomic DNA from both Pacific Lamprey and *Lampetra* spp, each assay amplified only the target lamprey, demonstrating specificity of the assays when samples contain both species. In addition, the presence of non-target lamprey DNA had no effect on the slope or efficiency of both qPCR assays (Table 2).

**Table 2:** Standard curve summaries. Pacific Lamprey (ETCytb_890-1015) and *Lampetra* spp (LACytb_890-1015) qPCR results for serial dilutions of target DNA (0.006 pg/μL–600 pg/μL) with and without 1 pg/μL of non-target DNA.

Assay	DNA	Slope	*R*^2^	Efficiency (%)	*Y*-intercept
ETCytb_890-1015	Pacific Lamprey	−3.58	0.992	90.23%	31.12
ETCytb_890-1015	Pacific Lamprey +*Lampetra* spp	−3.51	0.982	92.66%	30.92
LACytb_890-1015	*Lampetra* spp	−3.57	0.997	90.43%	31.00
LACytb_890-1015	*Lampetra* spp + Pacific Lamprey	−3.55	0.993	91.25%	31.04

Quantitative PCR on genomic DNA from lamprey distributed across Pacific coast drainages of North America indicated the assays were highly specific to the target species (Table 3, Table S2). The range in *C*_*t*_ values for Pacific Lamprey sampled within the Puget Sound watershed was 21.64–27.22. No Pacific Lamprey sampled from outside Puget Sound exceeded the maximum *C*_*t*_ value observed within Puget Sound. For *Lampetra* spp, the range in *C*_*t*_ values from samples collected within the Puget Sound watershed was 19.43–26.97. One *Lampetra* spp sampled from outside the Puget Sound (Toppenish Creek, WA, *C*_*t*_ = 27.18) slightly exceeded the maximum *C*_*t*_ value observed within Puget Sound. None of the 37 non-target fish species commonly found in the Pacific Northwest amplified with either assay (Table S2).

**Table 3:** Specificity of lamprey qPCR assays. Summary of sequence specificity and successful qPCR amplifications for the Pacific Lamprey (ETCytb_890-1015) and *Lampetra* spp (LACytb_890-1015) qPCR assay across *Entosphenus* spp and *Lampetra* spp.

	Pacific Lamprey assay (ETCytb_890-1015)	*Lampetra* spp assay (LACytb_890-1015)
Perfect sequence match across primers and probe	*E. tridentatus E*. *macrostomus E*. *minimus E*. *similis*	*L*. *ayresii L*. *richardsoni L*. *pacifica L*. *hubbsi Lampetra* spp (individuals identified only to genus)
Imperfect sequence match across primers and/or probe	*E*. *lethophagus*	*L*. *richardsoni* (Hunter Creek, CA; McGarvey Creek, CA; and Big River, WA) *Lampetra* spp (North Fork Suislaw River, OR; Kelsey Creek, CA; Mark West Creek, CA)
Successful amplification of template DNA	*E. tridentatus* (Nass River, BC; Buck Creek, BC; Stamp River, BC; Middle Fork Salmon River, ID; Middle Fork Clearwater River, ID; Lower Columbia River, WA; Entiat River, WA; Wenatchee River, WA; Black River, WA; Newaukum River, WA; Skookumchuck River, WA; Wind River, WA; Duckabush River, WA; Green River, WA; Nisqually River, WA; Dungeness River, WA; Shitike Creek, OR; Warm Springs River, OR; Pistol River, OR; Gualala River, CA; Feather River, CA; Arroyo Seco, CA)	*L*. *hubbsi* (Merced River, CA) *L. ayresii* (Sacramento River, CA) *L. richardsoni* (Farragut River, AK; Smith Creek, BC; Klickitat River, WA; Rock Creek, WA; Yakima River, WA; Toppenish Creek, WA; Black River, WA; Skookumchuck River, WA; Mosier Creek, OR; Euchre Creek, CA; Prairie Creek, CA) *Lampetra* spp (Green River, WA; Nisqually River, WA; Skagit River, WA; Puyallup River, WA; Tahuya River, WA; Snoqualmie River, WA; Nooksack River, WA)

All Pacific Lamprey cytb sequences (11 obtained from GenBank and 13 from our study) had 100% match with the target primers and the Pacific Lamprey specific probe (Fig. S2). The DNA sequence from *E*. *macrostomus*, *E*. *minimus*, and *E*. *similis* also had a complete match to the Pacific Lamprey primer and probe sequences, and *E*. *lethophagus* had one mismatch in the forward primer (Table 3). All *Entosphenus* spp had 5 mismatches with the *Lampetra* spp probe.

Across the set of *Lampetra* spp cytb sequence data (141 obtained from Genbank and 23 from our study), 85.4% completely matched the target primers and *Lampetra* specific probe (Table 3, Fig. S2). Specifically, 97.6% of *Lampetra* spp sequences had a complete match with the forward primer, 88.4% had complete match with the reverse primer, and 94.5% had complete match with the *Lampetra* spp probe. The *Lampetra* spp that mismatched with the primers and/or probe were from six locations: Hunter Creek, CA (five individuals sequenced and each had a single mismatch with the reverse primer); McGarvey Creek, CA (five individuals sequenced and each had a single mismatch with the reverse primer); Big River, WA (four individuals sequenced and one had a single mismatch with the reverse primer); North Fork Suislaw River, OR (five individuals sequenced and each had a single mismatch with the probe); Kelsey Creek, CA (four individuals sequenced and each had two mismatches with the forward primer and three mismatches with the reverse primer); and Mark West Creek, CA (four individuals sequenced and each had two mismatches with the reverse primer and two mismatches with the probe) (Table 3). The *Lampetra* from Hunter Creek, McGarvey Creek, and Big River are considered as *L*. *richardsoni* and group into the polytomy identified in Boguski et al. (2012), while the *Lampetra* from North Fork Suislaw River, Kelsey Creek and Mark West Creek represent genetically divergent populations. All *Lampetra* spp had five mismatches with the Pacific Lamprey probe, with the exception of the four individuals from Mark West Creek, CA, which had three mismatches.

### Detection of lamprey eDNA in Puget Sound watersheds

*Lampetra* spp and Pacific Lamprey eDNA was not detected in filtration controls, negative extraction controls and no template controls, demonstrating that there was no contamination during field or laboratory procedures.

**Figure 2:**
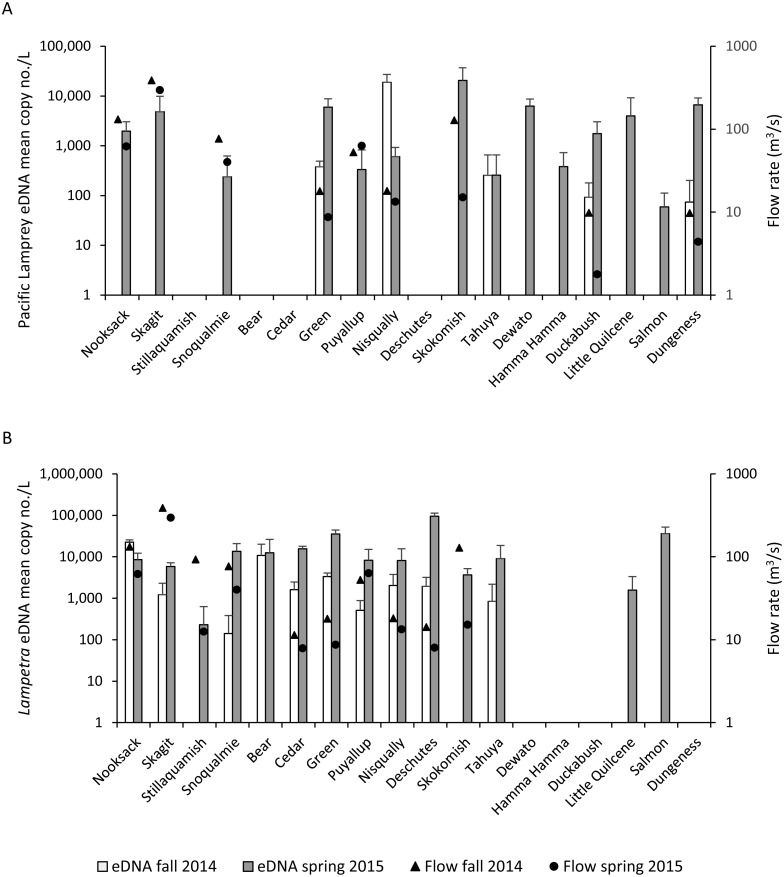
Lamprey eDNA concentration and stream flow. Mean copy number per L (copy no./L) (SD) of (A) Pacific Lamprey and (B) *Lampetra* spp eDNA at each sampling location and mean stream flow rate (fall = triangles, spring = circles) on days when water samples were collected during the fall 2014 and spring 2015.

Pacific Lamprey eDNA was detected in 14 of the 18 Puget Sound watersheds that were surveyed (Table 4, Fig. 2A). Five watersheds sampled in the fall and 14 watersheds sampled in the spring tested positive for Pacific Lamprey eDNA. All watersheds that tested positive for Pacific Lamprey in the fall also tested positive in spring. The Pacific Lamprey eDNA detection rate was 0.26 and 0.90 in the fall and spring, respectively, across the 14 sites where Pacific Lamprey eDNA was detected. Of the five locations where Pacific Lamprey eDNA was detected in both fall and spring, the mean eDNA concentration was higher in the fall for only the Nisqually River collection and was not different between fall and spring for the Tahuya River collection (Fig. 2A). The distribution of Pacific Lamprey in Puget Sound streams inferred through eDNA sampling was concordant with trap surveys (Hayes et al., 2013) for 16 of 18 watersheds (Table 4). We detected Pacific Lamprey eDNA in two streams (Skagit River and Snoqualmie River) where they were not observed by Hayes et al. (2013).

**Table 4:** Lamprey eDNA survey results across Puget Sound watersheds. The number of qPCRs (out of 12 qPCRs) above the limit of detection (LOD), the number of positive field replicates (out of three field replicates) during the fall 2014 and spring 2015 eDNA collections, and fish trap survey results from Hayes et al. (2013) (+, detected; −, not detected) at each sample location in Puget Sound watersheds. Bold text indicate seasonal mismatches in occupancy status between fall and spring sampling.

Watershed	Pacific Lamprey Hayes et al. (2013)	Pacific Lamprey qPCR results		*Lampetra* spp Hayes et al. (2013)	*Lampetra* spp qPCR results	
		Fall 2014	Spring 2015		Fall 2014	Spring 2015
Nooksack	+	**0/12; 0/3**	**12/12; 3/3**	+	11/12; 3/3	10/12; 3/3
Skagit	−	**0/12; 0/3**	**8/12; 3/3**	+	6/12; 2/3	12/12; 3/3
Stillaguamish	−	0/12; 0/3	0/12; 0/3	+	**0/12; 0/3**	**2/12; 1/3**
Snoqualmie	−	**0/12; 0/3**	**3/12; 2/3**	+	3/12; 1/3	12/12; 3/3
Bear	−	0/12; 0/3	1/12; 1/3 (0/12; 0/3)^a^	+	12/12; 3/3	11/12; 3/3
Cedar	−	0/12; 0/3	0/12; 0/3	+	12/12; 3/3	11/12; 3/3
Green	+	10/12; 3/3	10/12; 3/3	+	12/12; 3/3	11/12; 3/3
Puyallup	+	**0/12; 0/3**	**2/12; 2/3**	+	5/12; 3/3	9/12; 3/3
Nisqually	+	12/12; 3/3	8/12; 3/3	+	10/12; 3/3	8/12; 2/3
Deschutes	−	0/12; 0/3	0/12; 0/3	+	11/12; 3/3	12/12; 3/3
Skokomish	+	**0/12; 0/3**	**11/12; 3/3**	+	**0/12; 0/3**	**11/12; 3/3**
Tahuya	+	6/12; 2/3	3/12; 2/3	+	4/12; 2/3	7/12; 3/3
Dewatto	+	**0/12; 0/3**	**11/12; 3/3**	−	0/12; 0/3	0/12; 0/3
Hamma Hamma	+	**0/12; 0/3**	**7/12; 3/3**	−	0/12; 0/3	0/12; 0/3
Duckabush	+	2/12; 2/3	8/12; 3/3	−	0/12; 0/3	0/12; 0/3
Little Quilcene	+	**0/12; 0/3**	**9/12; 3/3**	+	**0/12; 0/3**	**6/12; 3/3**
Salmon	+	**0/12; 0/3**	**2/12; 2/3**	−	**0/12; 0/3**	**12/12; 3/3**
Dungeness	+	3/12; 1/3	12/12; 3/3	−	0/12; 0/3	0/12; 0/3

**Notes.**

The criteria for inferring species presence at a specific location at one sampling period (fall or spring) was a minimum of two qPCR amplifications exceeding the LOD, or one qPCR amplification exceeding the LOD in the original sample analysis and at least one qPCR amplification exceeding the LOD in a repeated sample analysis.

a Values in parentheses indicate repeated sample analysis.

*Lampetra* spp eDNA was detected in 10 watersheds in the fall and 14 watersheds in the spring (Table 4, Fig. 2B). The 10 watersheds where *Lampetra* spp were detected in the fall also tested positive in the spring. The *Lampetra* spp eDNA detection rate was 0.62 and 0.93 in the fall and spring, respectively, across the 14 sites where *Lampetra* spp eDNA was detected. Within watershed, *Lampetra* spp mean eDNA concentrations were higher on the spring collection day than on the fall collection day, with the exception of the Nooksack River (Fig. 2B). The distribution of *Lampetra* spp inferred through eDNA sampling was concordant with trap surveys (Hayes et al., 2013) for 17 of 18 watersheds (Table 4). We detected *Lampetra* spp eDNA in Salmon Creek, but individuals were not observed in juvenile salmon trapping operations (Hayes et al., 2013).

On days when water samples were collected, median stream flow was higher in the fall compared to spring for 11 of the 12 watersheds with USGS stream flow gages (Fig. 2, Table S3). The Puyallup River was the only watershed where the median stream flow was higher on the spring collection day compared to the fall collection day.

## Discussion

We developed efficient and robust qPCR assays that differentiate Pacific Lamprey and *Lampetra* spp across much of their range on the North American west coast (but see Assay limitations, below). We demonstrated the effectiveness of these assays for detecting eDNA from target species by surveying for Pacific Lamprey and *Lampetra* spp across Puget Sound watersheds in the fall and spring and found that detection of lamprey largely differed between seasons. These assays will be a valuable tool for resource managers and have application to lamprey conservation, including monitoring translocation and re-introduction programs, tracking recolonization following barrier removal projects, and mapping species distributions.

### Lamprey eDNA in Puget Sound watersheds

The distribution of lamprey in Puget Sound watersheds identified through eDNA sampling was largely consistent with the distribution observed during incidental trap catch surveys in 2011 (Hayes et al., 2013). For example, we detected eDNA from target species in all watersheds where target species were found by Hayes et al. (2013). This finding is not unexpected, as results from eDNA presence/absence surveys are often consistent with results from traditional field survey methods, such as electrofishing and trapping (Baldigo et al., 2017; McKelvey et al., 2016; Rees et al., 2014a). However, the traps used by Hayes et al. (2013) may under-report *Lampetra* spp that do not undergo active downstream migration (i.e., Western Brook Lamprey) relative to anadromous lamprey (i.e., Pacific Lamprey and Western River Lamprey). Using eDNA, we detected Pacific Lamprey in the Skagit and Snoqualmie rivers and *Lampetra* spp in Salmon Creek where individuals were not observed by Hayes et al. (2013). While Pacific Lamprey are known to inhabit the Skagit River watershed (Goodman et al., 2008) and the Snohomish River watershed (J Whitney, Washington Department of Fish and Wildlife, pers. comm., 2017), which includes the Snoqualmie River, *Lampetra* spp in Salmon Creek were heretofore unreported. Thus our findings provide a more complete picture for lamprey distributions in Puget Sound watersheds.

Detection of lamprey eDNA differed between seasons, highlighting the importance of considering sample timing when developing an eDNA sampling strategy. For example, across the fall sample collections, we failed to detect Pacific Lamprey at nine locations and *Lampetra* spp at four locations where each was detected in the spring sample collections. This finding is significant because larval lamprey have an extended freshwater rearing phase and should be present in these streams year-round, suggesting that eDNA should have been consistently detected. Furthermore, seasonality appeared to have a greater effect on Pacific Lamprey eDNA detections compared to *Lampetra* spp. For example, the eDNA detection rate for Pacific Lamprey was 3.5 times higher in spring while the eDNA detection rate for *Lampetra* spp was 1.5 times higher in spring. Studies that included a temporal sampling scheme have demonstrated that eDNA abundance varies over time (Buxton et al., 2017; De Souza et al., 2016; Furlan et al., 2016; Laramie, Pilliod & Goldberg, 2015; Spear et al., 2015), which could have important conservation implications that inform seasonal activities, such as life history events, migratory behaviors, and dispersal of invasive species. Although sampling in the fall did not provide additional information on lamprey distribution in Puget Sound, it revealed that certain time periods yield better detections than others, which can ultimately influence cost effective sampling strategies for target organisms.

The difference in eDNA detection between the fall and spring samples within watersheds was likely due, in part, to different stream flow rates between seasons. Within watersheds, stream flow rates were typically higher when we sampled in the fall and coincided with lower observed eDNA concentrations. During periods of higher flows, cellular material shed from organisms may be diluted (Gingera et al., 2016; Jane et al., 2015), resulting in lower eDNA concentrations per sample volume or false-negative detections if eDNA concentrations fall below the LOD threshold required for a positive detection. The relationship between eDNA concentration and stream flow rate is complex because the fate of eDNA in aquatic environments is also influenced by a suite of environmental (Jane et al., 2015; Lacoursiere-Roussel, Rosabal & Bernatchez, 2016; Moyer et al., 2014; Pilliod et al., 2014; Strickler, Fremier & Goldberg, 2015), demographic (Diaz-Ferguson et al., 2014; Takahara et al., 2012; Wilcox et al., 2016), and biological (De Souza et al., 2016; Maruyama et al., 2014) factors. Further studies will be required in order to quantify relationships between lamprey eDNA abundance and flow rates.

Most of our results support the conclusion that eDNA abundance of target organisms was lower during periods of higher flow; however, a few of our results stand in contrast. For example, Pacific Lamprey eDNA in the Nisqually River and *Lampetra* spp eDNA in the Nooksack River was more concentrated on the fall sample collection date, coinciding with a higher stream flow rate. Furthermore, Pacific Lamprey and *Lampetra* spp eDNA in the Puyallup River was more concentrated on the spring sample collection date (assuming larval Pacific Lamprey were present year-round), despite a higher stream flow in the Puyallup River on the spring collection date. If stream flow was the only factor that affected eDNA abundance within watershed, then we would expect eDNA to be more concentrated on the collection date that had a lower stream flow rate. Our findings suggest that factors other than stream flow may have affected eDNA abundance.

Seasonal life history events may also have influenced lamprey eDNA abundance. Here, we address two such seasonal life history events, spawning and downstream movement of larvae and juveniles. Spawning activity and decomposition of dead adults in semelparous species would be expected to increase shedding of cells and tissues into aquatic environments. Further, increased eDNA abundance during reproductive seasons has been observed for other aquatic species, including Sea Lamprey (*Petromyzon marinus*) (Gingera et al., 2016), Eastern Hellbender (*Cryptbranchus alleganiensis*) (Spear et al., 2015), and Great Crested Newt (*Triturus cristatus*) (Buxton et al., 2017). We had limited information on spawn timing within our study sites, however, we observed dead *Lampetra* spp adults in the Bear Creek and Cedar River traps when water samples were collected in spring. In addition, higher abundance of lamprey eDNA during the spring sample collection appeared to coincide with spawn timing reported from other locations (Beamish, 1980; Stone, 2006). For example, in several streams (e.g., Dewatto River), no water samples tested positive for lamprey eDNA in the fall, whereas all three water samples tested positive in the spring. Thus, increased eDNA abundance in spring samples may be attributed to adults through the release of gametes, shedding of cells during nest building behavior, and decomposition of adults. Larval and juvenile lamprey both exhibit downstream movement. Larvae disperse downstream in late winter through spring (Potter, 1980) and juveniles outmigrate from fall through spring (Beamish & Levings, 1991; Richards & Beamish, 1981). Interestingly, the majority of lamprey captured in fish traps in the study by Hayes et al. (2013) (which was conducted mostly in April through June) were larvae, suggesting that downstream movement of larvae occurs in the Puget Sound watershed that we sampled. Downstream movement requires lamprey to emerge from sediments and enter the water column and if such movement was common in spring, but not fall, it could contribute to seasonal differences in eDNA abundance. Temporal sampling of water in fall, winter and spring could provide information on the timing of downstream movements and outmigration.

### Inferring species presence

Presently, there are no criteria regarding the minimum number of positive PCRs that are required for inferring species presence. eDNA studies have typically required a minimum of one positive (Laramie, Pilliod & Goldberg, 2015; McKelvey et al., 2016) or more than one positive (Rees et al., 2014a) out of several PCRs performed on a sample site. Furthermore, repeat PCRs are often performed on field replicates that yielded ambiguous results (Goldberg et al., 2013; Laramie, Pilliod & Goldberg, 2015; Rees et al., 2014a), with the aim of improving confidence in results. Goldberg et al. (2016) recommend that caution be used when inferring species presence at sample sites where only one of several PCR replicates tests positive for the target species and where the results were not replicated through repeat analysis of samples or repeated site visits. We followed this recommendation for inferring species presence and required that at least two qPCRs exceed the LOD, or when only one qPCR exceeded the LOD we required replication of this result in a repeat analysis of samples. Our criteria affected the inference of Pacific Lamprey at one location, Bear Creek, where only one of 12 qPCRs from the spring collection day and no qPCRs from the fall collection day exceeded the LOD. Repeat analysis on Bear Creek spring samples yielded no positive qPCRs. Under our criteria, the results for Bear Creek imply non-detection of Pacific Lamprey. However, application of less stringent criteria for inferring species presence, such as requiring that only a single qPCR exceed the LOD, would result in a different conclusion. Nevertheless, our goal was to apply a conservative approach for inferring species presence and our findings may warrant further evaluation for the presence of Pacific Lamprey in Bear Creek.

### Application of LNAs to eDNA studies

Here, we included locked nucleic acids (LNAs) in the Taqman assay probe designs. Few studies to date have applied LNA-based PCR assays to environmental samples and these studies have generally been limited to detecting pathogens isolated from host tissues (Barkallah et al., 2016; Ruthig & DeRidder, 2012; Yamada et al., 2008), raw sewage (Alonso, Amoros & Canigral, 2011), and soil (Irenge et al., 2010). Locked nucleic acids offer an alternative chemistry for robust PCR assay design that is compatible with DNA chemistry. Therefore, LNA bases can be incorporated into primer and probe sequences allowing LNA-DNA mixmer oligonucleotides to be designed (Mouritzen et al., 2003). Several key features render LNAs well suited for eDNA assay design, including high sensitivity (Alonso, Amoros & Canigral, 2011; Josefsen et al., 2009; Letertre et al., 2003), high specificity (Letertre et al., 2003; Owczarzy et al., 2011; You et al., 2006), and good performance with low DNA concentrations (Ballantyne, Van Oorschot & Mitchell, 2008; Reynisson et al., 2006). Furthermore, LNA probes perform well in Taqman assays, as applied in our study, and produce results that are comparable to MGB probes (Letertre et al., 2003; Ruthig & DeRidder, 2012). Conserved forward primers and nearly conserved reverse primers between Pacific Lamprey and *Lampetra* spp LNA assays stands in contrast to the convention for MGB assays where assay specificity was found to be largely influenced by mismatches in the primers (Wilcox et al., 2013). Placement of LNAs into optimal and strategic positions increases the *T*_*m*_ (temperature at which half of double stranded DNA has dissociated into single strands). However, thermal stability of LNA-DNA duplexes and mismatch discrimination depends on the position of LNA bases in a sequence, the number of LNAs in sequence, and specific bases that are modified (Owczarzy et al., 2011; You et al., 2006). Thus, LNA technology has application toward eDNA assay design and LNAs may be particularly effective for differentiating closely related taxa where a limited set of base pair mismatches may constrain assay design.

### Assay limitations

While the ETCytb_890-1015 and LACytb_890-1015 assays differentiated Pacific Lamprey and *Lampetra* spp, there are limitations to each assay that need to be considered (Table 3). Sequence comparison of ETCytb_890-1015 target sites among *Entosphenus* spp showed that primer and probe sites are conserved among Vancouver Lamprey (*E. macrostomus*, distributed in the Lake Cowichan drainage, Vancouver Island, BC), Miller Lake Lamprey (*E*. *minimus*, distributed in the Upper Klamath River drainage, OR), Klamath Lamprey (*E*. *similis*, distributed in the Klamath River drainage, OR and CA), and Pit-Klamath Brook Lamprey (*E*. *lethophagus*, distributed in the Klamath River drainage, OR and CA, and Pit River drainage, CA) (*Entosphenus* spp distributions from Potter et al., (2015)), with the exception that the forward primer has one mismatch with Pit-Klamath Brook Lamprey. Similar findings of conserved primer and probe sites among *Entosphenus* spp were reported for an eDNA assay that was developed for Pacific Lamprey in the Columbia River based on cytochrome c oxidase I (COI), with the exception that the Pit-Klamath Brook Lamprey sequence was conserved with the COI primer and probe sites and Vancouver Lamprey and Miller Lake Lamprey sequences were not available for comparison (Carim et al., 2017). It is unknown how the single mismatch in the ETCytb_890-1015 forward primer would affect amplification of Pit-Klamath Brook Lamprey DNA.

The LACytb_890-1015 assay detects *L*. *ayresii*, *L*. *richardsoni*, *L*. *pacifica*, and *L*. *hubbsi*, although the assay may have limited or no detectability of *Lampetra* populations in certain geographic locations (Table 3). These *Lampetra* populations were represented by *L. richardsoni* in Hunter Creek, CA, McGarvey Creek, CA, and Big River, WA, and genetically divergent *Lampetra* spp in North Fork Siuslaw River, OR, Kelsey Creek, CA, and Mark West Creek, CA. Boguski et al. (2012) suggested the *Lampetra* spp in Siuslaw River, Kelsey Creek, and Mark West Creek could represent cryptic and previously undescribed species. Because the understanding of genetic diversity in *Lampetra* spp is far from complete, it is possible that some unsampled *Lampetra* populations, particularly populations south of the Columbia River Basin, may be found containing mismatches to the LACytb_890-1015 assay. Further testing of genomic DNA on mismatched sequences would provide information on the applicability of the *Lampetra* spp assay for detecting eDNA at locations where individuals lack complete primer and probe match.

The ETCytb_890-1015 and LACytb_890-1015 assays are each genus-specific, but these assays may be used to infer species presence when distributional information is taken into account. For example, the ETCytb_890-1015 assay may be applied to several *Entosphenus* spp and the resolution of detection to species or genus would depend on whether one or more than one *Entosphenus* spp, respectively, is present in a particular watershed. The overlap in geographic distribution between Pacific Lamprey and other *Entosphenus* spp is limited; Pacific Lamprey is broadly distributed whereas *E*. *macrostomus*, *E*. *minimus*, *E*. *similis*, *E*. *lethophagus* have restricted geographic distributions. Consequently, Pacific Lamprey occur in the absence of other *Entosphenus* spp throughout most of the Pacific Lamprey range. Thus, eDNA may be resolved specifically to Pacific Lamprey across a large portion of their range (geographic regions where Pacific Lampey is the only known *Entosphenus* spp present) and resolved more generally to *Entosphenus* spp across a small portion of the Pacific Lamprey range (geographic regions where Pacific Lampey and other *Entosphenus* spp overlap in distribution). Likewise, distributional information on *Lampetra* spp could also be incorporated when resolving detections between genus and species. In geographic regions where *Lampetra* spp overlap in distribution, resource managers may simply want information on occupancy status of *Lampetra* spp, in general.

## Conclusions

Knowledge of the distribution and occupancy patterns of native lamprey in Pacific coast drainages of North America is needed for advancing conservation goals (Clemens et al., 2017; Luzier et al., 2011; Wang & Schaller, 2015). Surveys that apply targeted approaches with lamprey-specific electrofishers have been shown to achieve high detection probability (>90%) for larval lamprey when these methods are employed at single sites with suitable larval rearing habitats (Reid & Goodman, 2015). Our eDNA assays provide an additional approach that resource managers can use to survey for lamprey. Interestingly, Reid & Goodman (2015) failed to detect Pacific Lamprey in the Hamma Hamma River, but Pacific Lamprey are known to occur in this river (Hayes et al., 2013) and we detected their eDNA. It is important to understand that eDNA-based methods, like other species detection methods, are sensitive to environmental conditions. For example, higher stream flows in the fall likely influenced the lack of lamprey eDNA detection in some streams, although larvae were probably present year-round. Lamprey eDNA detection could also be improved by sampling water at habitat patches containing the substrate type preferred by larvae, an approach used by Reid & Goodman (2015) for achieving high detection probabilities with electrofishing. Furthermore, because larval lamprey typically reside in sediments for 3–7 years (Dawson et al., 2015), eDNA sampled from sediments could potentially identify habitat patches that are occupied. Lastly, while our assays differentiate Pacific Lamprey and *Lampetra* spp across a large geographic region, we recommend testing qPCR amplification on individuals prior to performing eDNA studies at locations that were not represented in our study.

##  Supplemental Information

10.7717/peerj.4496/supp-1 Table S1 Accession number and location in Puget Sound, Washington, for the 13 Pacific Lamprey and 23 *Lampetra* spp that were used to develop the qPCR assays

10.7717/peerj.4496/supp-2 Table S2 Raw data from qPCR assay specificity testing Quantitative pPCR summary results for 91 Pacific Lamprey and 59 *Lampetra* spp distributed from Alaska to California and 37 fish species common to Puget Sound watersheds that were tested against the ETCytb_890-1015 and LACytb_890-1015 qPCR assays. Ct values are provided for samples that amplified a PCR product (X = no amplification).

10.7717/peerj.4496/supp-3 Table S3 Stream sampling information Date of water sample collections, median 24 hour stream flow (m^3^/s) recorded during the fall 2014 and spring 2015 water sampling date for each Puget Sound watershed with a stream flow gage station, and the site locations (latitude and longitude) of the traps that were monitored by Hayes et al. (2013).

10.7717/peerj.4496/supp-4 Figure S1 Limit of detection (LOD) and limit of quantification (LOQ) for the PacificLamprey (ETCytb_890-1015) and *Lampetra* spp (LACytb_890-1015) qPCR assays The limits were estimated by performing qPCR on a dilution series of each target oligonucleotide ultramer (1, 2, 3, 6, 30, 60, 300, and 600 copies/qPCR), with eight qPCR replicates per concentration. LOD is the minimum number of copies that could be detected and LOQ is the lowest number of copies that yielded positive amplification across the eight replicates.

10.7717/peerj.4496/supp-5 Figure S2 Pacific Lamprey (ETCytb_890-1015) and *Lampetra* spp (LACytb_890-1015) target sequence Comparison of *Entosphenus* spp (*N* = 28 total) and * Lampetra* spp (*N* = 164 total) mtDNA sequence data across 126 contiguous bases in the * cytb* region for which qPCR assays were developed in this study. Sequence data are from our study, Boguski et al. (2012), Reid et al. (2011), Lang et al. (2009). Primers are highlighted in grey, the Pacific Lamprey probe is highlighted in yellow, and the * Lampetra* spp probe is highlighted in green. Samples (GenBank accession numbers) with mismatched nucleotides are shown within collection site.

10.7717/peerj.4496/supp-6 Data S1 Raw qPCR data from Pacific Lamprey and *Lampetra* spp eDNA surveys of 18 Puget Sound watersheds, stream flow, and sampling location (latitude, longitude)

## Additional Information and Declarations

### DNA Deposition

The following information was supplied regarding the deposition of DNA sequences: The new cytochrome b sequence from this study is accessible via Genbank accession numbers KU672473–KU672508.

### Data Availability

The following information was supplied regarding data availability: The raw data are available as Data S1, and are housed in ScienceBase. Citation: Ostberg CO, Chase DM, Hayes MC, Duda JJ. Distribution and seasonal differences in Pacific Lamprey and *Lampetra* spp eDNA across 18 Puget Sound watersheds, 2014 and 2015: U.S. Geological Survey data release, DOI 10.5066/F7H994DT.
