# Supplementary material for: Distribution and seasonal differences in Pacific Lamprey and Lampetra spp eDNA across 18 Puget Sound watersheds
Source: PeerJ. 2018 Mar 16;6:e4496. doi: 10.7717/peerj.4496 (PMC5858536; doi:10.7717/peerj.4496)
Supplement: Table S1 [file peerj-06-4496-s001.docx]

| Accession^1^# | Location (river) | Morphological assignment^2^ | *cytb* RFLP assay assignment^2^ |
| --- | --- | --- | --- |
| KU672473 | Duckabush | Pacific Lamprey | Pacific Lamprey |
| KU672474 | Dungeness | Pacific Lamprey | Pacific Lamprey |
| KU672475 | Dungeness | Pacific Lamprey | Pacific Lamprey |
| KU672476 | Dungeness | Pacific Lamprey | Pacific Lamprey |
| KU672477 | Dungeness | Pacific Lamprey | Pacific Lamprey |
| KU672478 | Dungeness | Pacific Lamprey | Pacific Lamprey |
| KU672479 | Dungeness | Pacific Lamprey | Pacific Lamprey |
| KU672480 | Green | Pacific Lamprey | Pacific Lamprey |
| KU672481 | Green | Pacific Lamprey | Pacific Lamprey |
| KU672482 | Green | Pacific Lamprey | Pacific Lamprey |
| KU672483 | Green | Pacific Lamprey | Pacific Lamprey |
| KU672484 | Green | Pacific Lamprey | Pacific Lamprey |
| KU672485 | Nisqually | Pacific Lamprey | Pacific Lamprey |
| KU672486 | Green | *Lampetra* | *Lampetra* |
| KU672487 | Nisqually | *Lampetra* | *Lampetra* |
| KU672488 | Nisqually | *Lampetra* | *Lampetra* |
| KU672489 | Nisqually | *Lampetra* | *Lampetra* |
| KU672490 | Nisqually | *Lampetra* | *Lampetra* |
| KU672491 | Nisqually | *Lampetra* | *Lampetra* |
| KU672492 | Nisqually | *Lampetra* | *Lampetra* |
| KU672493 | Nisqually | *Lampetra* | *Lampetra* |
| KU672494 | Nisqually | *Lampetra* | *Lampetra* |
| KU672495 | Nisqually | *Lampetra* | *Lampetra* |
| KU672496 | Nisqually | *Lampetra* | *Lampetra* |
| KU672497 | Nisqually | *Lampetra* | *Lampetra* |
| KU672498 | Nisqually | *Lampetra* | *Lampetra* |
| KU672499 | Nooksack | *Lampetra* | *Lampetra* |
| KU672500 | Puyallup | *Lampetra* | *Lampetra* |
| KU672501 | Puyallup | *Lampetra* | *Lampetra* |
| KU672502 | Puyallup | *Lampetra* | *Lampetra* |
| KU672503 | Skagit | *Lampetra* | *Lampetra* |
| KU672504 | Skagit | *Lampetra* | *Lampetra* |
| KU672505 | Snoqualmie | *Lampetra* | *Lampetra* |
| KU672506 | Tahuya | *Lampetra* | *Lampetra* |
| KU672507 | Tahuya | *Lampetra* | *Lampetra* |
| KU672508 | Tahuya | *Lampetra* | *Lampetra* |

^1^All samples were from juveniles, with the exception of KU672499, KU672500, and KU672505 which were from adults.

^2^Results from Hayes et al. 2013.
