# Supplementary material for: Distribution and seasonal differences in Pacific Lamprey and Lampetra spp eDNA across 18 Puget Sound watersheds
Source: PeerJ. 2018 Mar 16;6:e4496. doi: 10.7717/peerj.4496 (PMC5858536; doi:10.7717/peerj.4496)
Supplement: Table S3 — Date of water sample collections, median 24 hour stream flow (m3/s) recorded during the fall 2014 and spring 2015 water sampling date for each Puget Sound watershed with a stream flow gage station, and the site locations (latitude and longitude) of the traps that were monitored by Hayes et al. (2013). [file peerj-06-4496-s003.docx]

|  |  |  | Fall 2014 | |  | Spring 2015 | |  |
| --- | --- | --- | --- | --- | --- | --- | --- | --- |
| Watershed | Latitude | Longtitude | Date | Median flow (m3/s) |  | Date | Median flow (m3/s) | Gage station |
| Nooksack | 48.83177 | 122.59810 | 10/21/2014 | 131.96 |  | 5/28/2015 | 62.01 | Ferndale |
| Skagit | 48.44554 | 122.32773 | 10/21/2014 | 389.36 |  | 5/28/2015 | 295.91 | Mount Vernon |
| Stillaguamish | 48.20590 | 122.26620 | 10/21/2014 | 92.74 |  | 5/28/2015 | 12.54 | Arlington |
| Snoqualmie | 47.72294 | 122.00758 | 10/21/2014 | 76.74 |  | 5/28/2015 | 40.21 | Carnation |
| Bear | 47.66960 | 122.11017 | 10/9/2014 | - |  | 6/29/2015 | - | No station^1^ |
| Cedar | 47.48602 | 122.20787 | 10/9/2014 | 11.41 |  | 6/29/2015 | 7.90 | Renton |
| Green | 47.29375 | 122.16773 | 10/9/2014 | 17.98 |  | 6/29/2015 | 8.69 | Auburn |
| Puyallup | 47.19759 | 122.25369 | 10/9/2014 | 52.67 |  | 5/6/2015 | 63.29 | Puyallup |
| Nisqually | 46.97429 | 122.63287 | 10/29/2014 | 18.12 |  | 6/29/2015 | 13.37 | McKenna |
| Deschutes | 47.01844 | 122.90381 | 10/29/2014 | 14.22 |  | 5/6/2015 | 8.01 | Tumwater |
| Skokomish^2^ | 47.31308 | 123.17983 | 11/5/2014 | 128.70 |  | 5/6/2015 | 15.15 | Potlach |
| Tahuya | 47.38954 | 123.02026 | 11/5/2014 | - |  | 6/30/2015 | - | No station^1^ |
| Dewatto | 47.45454 | 123.04589 | 11/5/2014 | - |  | 6/30/2015 | - | No station^1^ |
| Hamma-Hamma | 47.55294 | 123.04240 | 11/13/2014 | - |  | 6/30/2015 | - | No station^1^ |
| Duckabush | 47.65482 | 122.94536 | 11/13/2014 | 9.83 |  | 6/30/2015 | 1.78 | Brinnon |
| Little Quilcene | 47.82785 | 122.86492 | 11/13/2014 | - |  | 6/30/2015 | - | No station^1^ |
| Salmon | 47.98524 | 122.89010 | 11/13/2014 | - |  | 6/30/2015 | - | No station^1^ |
| Dungeness | 48.14857 | 123.12617 | 11/13/2014 | 9.80 |  | 6/30/2015 | 4.39 | Sequim |

^1^Watershed without a stream flow gage station.

^2^Stream flow data on 11/5/2014 for the Skokomish River were missing from the USGS stream gage data base, so the flow on 11/6/2014 was used in its place.
