# Supplementary material for: Distribution and seasonal differences in Pacific Lamprey and Lampetra spp eDNA across 18 Puget Sound watersheds
Source: PeerJ. 2018 Mar 16;6:e4496. doi: 10.7717/peerj.4496 (PMC5858536; doi:10.7717/peerj.4496)

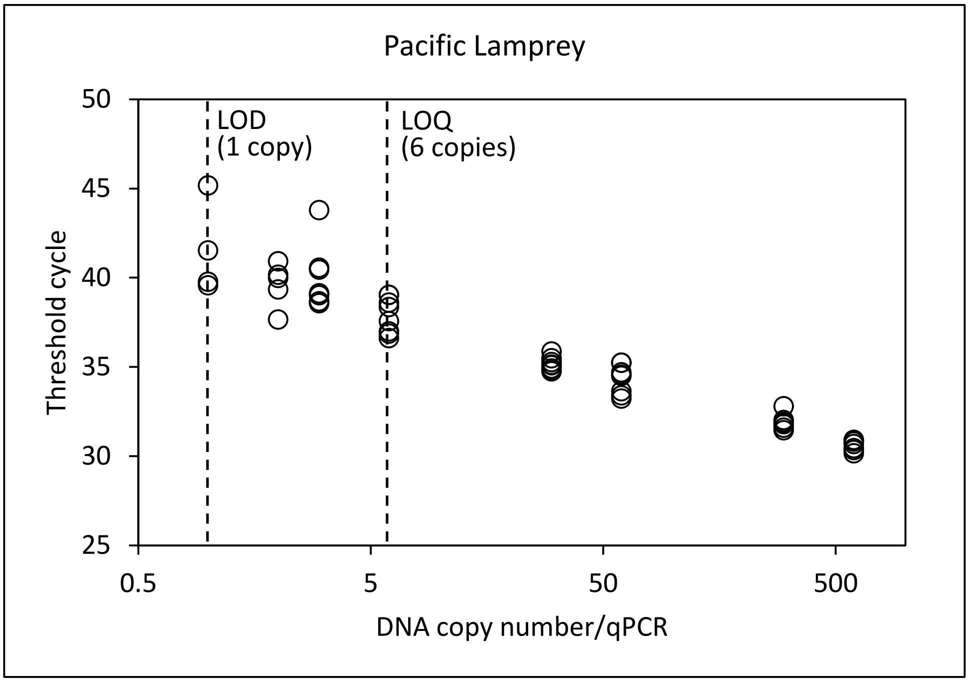
Pacific Lamprey
50
45
40
35
30
25
Threshold cycle
LOD
(1 copy)
LOQ
(6 copies)
0.5
5
50
500
DNA copy number/qPCR

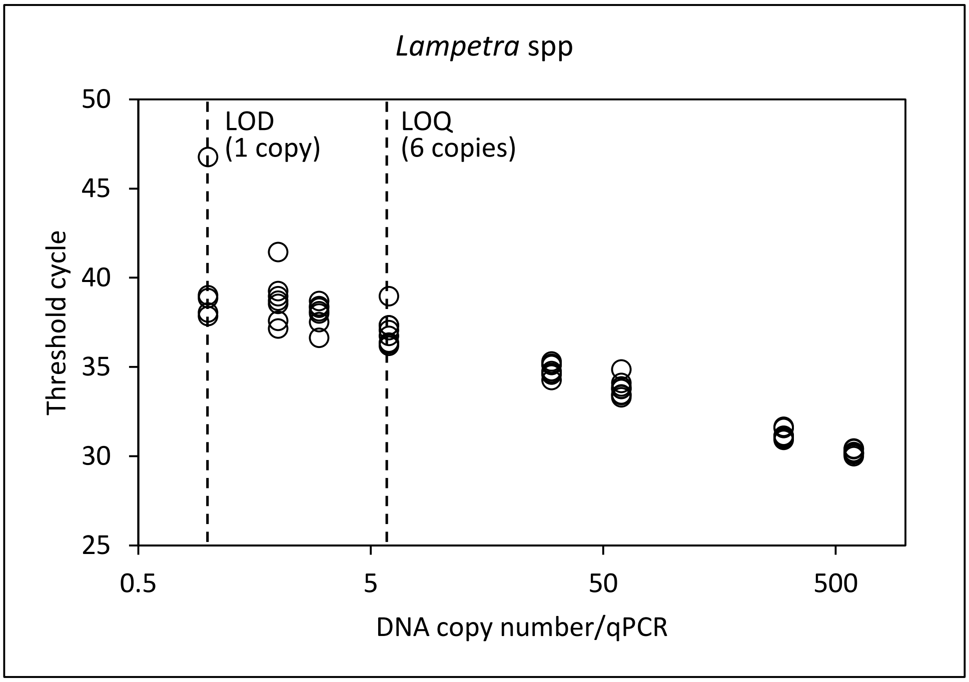
Lampetra spp
50
45
40
35
30
25
Threshold cycle
LOD
(1 copy)
LOQ
(6 copies)
0.5
5
50
500
DNA copy number/qPCR

Figure S1. Limit of detection (LOD) and limit of quantification (LOQ) for the Pacific Lamprey (ETCytb_890-1015) and *Lampetra* spp (LACytb_890-1015) qPCR assays developed in this study. The limits were estimated by performing qPCR on a dilution series of each target oligonucleotide ultramer (1, 2, 3, 6, 30, 60, 300, and 600 copies/qPCR), with eight qPCR replicates per concentration. LOD is the minimum number of copies that could be detected and LOQ is the lowest number of copies that yielded positive amplification across the eight replicates.

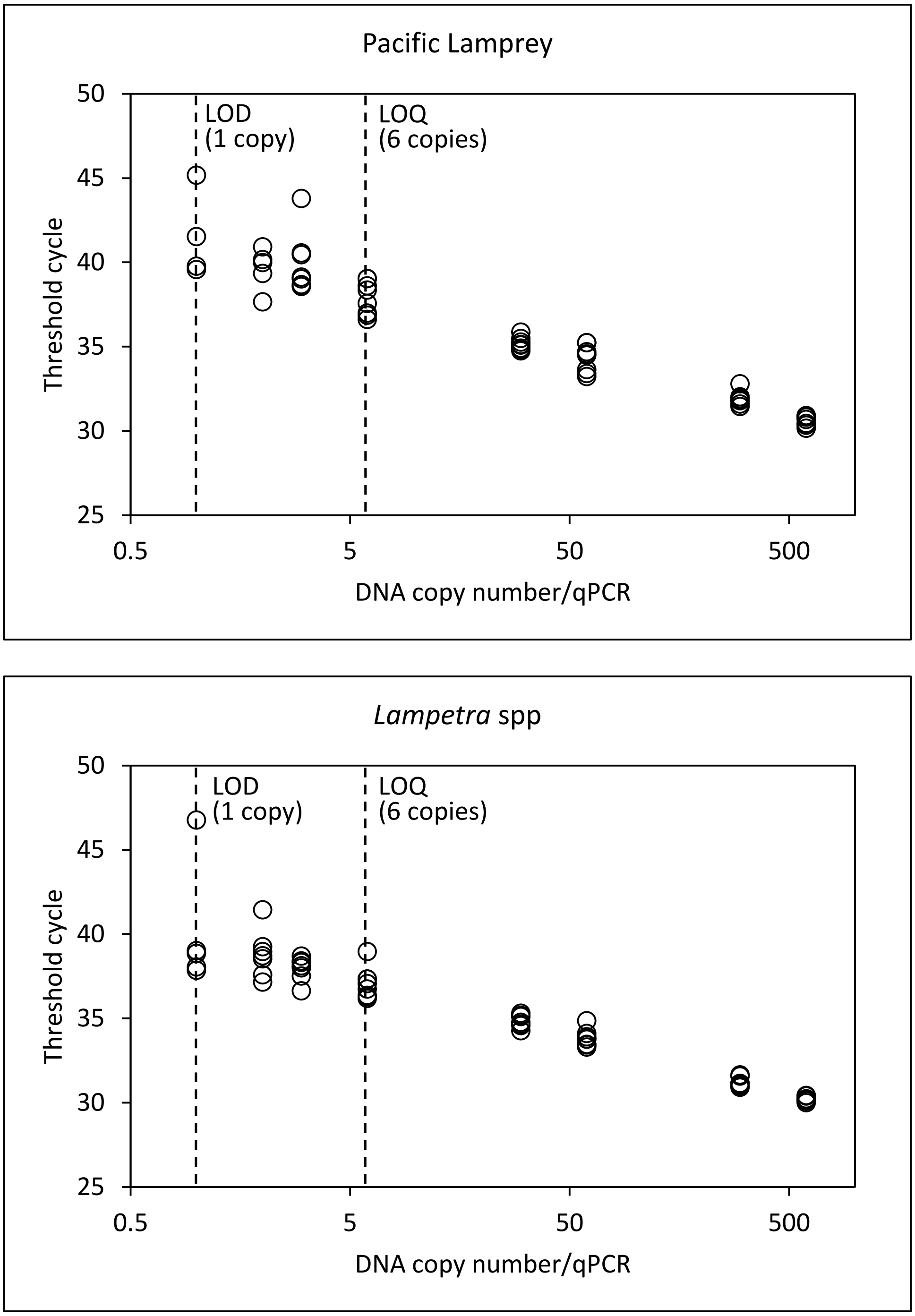

Supplement: Figure S1 — The limits were estimated by performing qPCR on a dilution series of each target oligonucleotide ultramer (1, 2, 3, 6, 30, 60, 300, and 600 copies/qPCR), with eight qPCR replicates per concentration. LOD is the minimum number of copies that could be detected and LOQ is the lowest number of copies that yielded positive amplification across the eight replicates. [file peerj-06-4496-s004.pdf]
