# Supplementary material for: Distribution and seasonal differences in Pacific Lamprey and Lampetra spp eDNA across 18 Puget Sound watersheds
Source: PeerJ. 2018 Mar 16;6:e4496. doi: 10.7717/peerj.4496 (PMC5858536; doi:10.7717/peerj.4496)
Supplement: Figure S2 — Comparison of Entosphenus spp (N = 28 total) and Lampetra spp (N = 164 total) mtDNA sequence data across 126 contiguous bases in the cytb region for which qPCR assays were developed in this study. Sequence data are from our study, Boguski et al. (2012), Reid et al. (2011), Lang et al. (2009). Primers are highlighted in grey, the Pacific Lamprey probe is highlighted in yellow, and the Lampetra spp probe is highlighted in green. Samples (GenBank accession numbers) with mismatched nucleotides are shown within collection site. [file peerj-06-4496-s005.pdf]

	1–42
E. tridentatus, N = 24	CTTTAGCAGCAGCCATCATAATCCTCCTAGTTATTCCATTTA
Lampetra spp, N = 140	......................T...........C..C....
Lampetra spp, Big River, WA (GU120746)	......................T...........C..C....
Lampetra spp, North Fork Suislaw River, OR (GU120813, GU120814, GU120815, GU120816, GU120817)	......................T...........C..C....
Lampetra spp, Hunter Creek, CA (GU120831, GU120832, GU120833, GU120834, GU120835)	......................T...........C..C....
Lampetra spp, McGarvey Creek, CA (GU120836, GU120837, GU120838, GU120839, GU120840)	......................T...........C..C....
Lampetra spp, Kelsey Creek, CA (GU120858, GU120859, GU120860, GU120861)	.................GC...T...........C..C....
Lampetra spp, Mark West Creek, CA (GU120862, GU120863, GU120865, GU120864)	......................T........A..C..C...G
E. lethophagus (GQ206153)	.......C..................................
E. macrostomus, *E. minimus*, and *E. similus*	....

	43–84
E. tridentatus, N = 24	CCCATACCTCTAAACAGCGTGGTATCCAATTCCGCCCACTTG
Lampetra spp, N = 140	....C...........A.....C..T.....T..T..G....
Lampetra spp, Big River, WA (GU120746)	....C...........A.....C..T.....T..T..G....
Lampetra spp, North Fork Suislaw River, OR (GU120813, GU120814, GU120815, GU120816, GU120817)	....C...........A.....C..T.....T..T..GT...
Lampetra spp, Hunter Creek, CA (GU120831, GU120832, GU120833, GU120834, GU120835)	....C...........A.....C..T.....T..T..G....
Lampetra spp, McGarvey Creek, CA (GU120836, GU120837, GU120838, GU120839, GU120840)	....C...........A.....C..T.....T..T..G....
Lampetra spp, Kelsey Creek, CA (GU120858, GU120859, GU120860, GU120861)	.T..C...........A.A...C..T.....T..T..G....
Lampetra spp, Mark West Creek, CA (GU120862, GU120863, GU120865, GU120864)	.T..C........G..A........T.....T..T.......
E. lethophagus (GQ206153)	....
E. macrostomus, *E. minimus*, and *E. similus*	....

	85–126
E. tridentatus, N = 24	CTCAAATTACATTTTGAATTCTAATCGCTGATCTAGCACTAC
Lampetra spp, N = 140	.C..........-C...........T................
Lampetra spp, Big River, WA (GU120746)	.C...........C...........T...........G....
Lampetra spp, North Fork Suislaw River, OR (GU120813, GU120814, GU120815, GU120816, GU120817)	.C..G...T....C...........T................
Lampetra spp, Hunter Creek, CA (GU120831, GU120832, GU120833, GU120834, GU120835)	.C...........C...........T..C.............
Lampetra spp, McGarvey Creek, CA (GU120836, GU120837, GU120838, GU120839, GU120840)	.C...........C...........T..C.............
Lampetra spp, Kelsey Creek, CA (GU120858, GU120859, GU120860, GU120861)	.C.......T...C...........T.....C....TT....
Lampetra spp, Mark West Creek, CA (GU120862, GU120863, GU120865, GU120864)	.C...........C....C......TA...............
E. lethophagus (GQ206153)	....
E. macrostomus, *E. minimus*, and *E. similus*	....
